# Plant Growth-Promoting Rhizobacteria as a Strategy to Enhance Enzymatic and Metabolic Tolerance of *Cucumis sativus* L. Under Salinity Stress

**DOI:** 10.3390/microorganisms14020351

**Published:** 2026-02-03

**Authors:** Laura-Andrea Pérez-García, Jorge Sáenz-Mata, Manuel Fortis-Hernandez, Pablo Preciado-Rangel

**Affiliations:** 1 Instituto Tecnológico de Torreón, Tecnológico Nacional de México, Carretera Torreón-San Pedro km 7.5, Torreón 27170, Coahuila, Mexico; manuel.fh@torreon.tecnm.mx; 2 Instituto Tecnológico de La Laguna, Tecnológico Nacional de México, Blvd. Revolución y, Av. Instituto Tecnológico de La Laguna s/n, Primero de Cobián Centro, Torreón 27000, Coahuila, Mexico; 3 Microbial Ecology Laboratory, Facultad de Ciencias Biológicas, Universidad Juárez del Estado de Durango, Av. Universidad s/n, Col. Filadelfia, Gómez Palacio 35010, Durango, Mexico; jsaenz_mata@ujed.mx

**Keywords:** bioestimulants, sustainable agriculture, salinity tolerance, PGPR

## Abstract

*Cucumis sativus* L., a salt-sensitive horticultural crop, is severely affected by soil salinity, which disrupts photosynthetic efficiency and metabolic homeostasis. This study quantified the effects of Plant Growth-Promoting Rhizobacteria (PGPR)—*Pseudomonas paralactis*, *Bacillus cereus*, *Sinorhizobium meliloti*, and *Acinetobacter radioresistens*—on key enzymatic indicators of cucumber seedlings exposed to 0, 50, 100, and 150 mM NaCl. PGPR inoculation significantly enhanced bacterial stress-mitigation and hormonal pathways, with ACC-deaminase activity increasing by up to 78.8% (*A. radioresistens*, 150 mM NaCl) and nitrilase activity by 50.5% (*S. meliloti*, 50 mM NaCl). Auxin-related pathways were strongly induced, as reflected by increases of up to 51.1% in the IAM pathway (*P. paralactis*) and 42.9% in the IPA pathway (*A. radioresistens*). In plant tissues, key metabolic enzymes exhibited high stability under salinity, with ProDH and NDPK activities increasing by up to 4.5% and 2.35%, respectively, while RuBisCO activity remained unaffected across treatments. These results demonstrate that PGPR function as effective bioestimulants by coordinating hormonal regulation and metabolic resilience, providing a sustainable biotechnological strategy to enhance cucumber tolerance to salinity stress.

## 1. Introduction

*Cucumis sativus* L. (cucumber) is one of the most economically important horticultural crops worldwide, extensively cultivated in temperate and tropical regions for fresh consumption and processing. Its high market demand and short production cycle make cucumber a key crop for protected and open-field agriculture. However, cucumber productivity is strongly constrained by adverse environmental conditions, particularly abiotic stresses [1]. Among these stresses, soil salinity represents a major limitation to cucumber cultivation, especially in arid and semi-arid regions where irrigation practices and climate change accelerate salt accumulation in agricultural soils. Soil salinization affects nearly 7% of the world’s land surface and over one-third of irrigated farmland, resulting in annual yield losses exceeding 27 billion USD [2,3].

Excess salts disrupt soil structure and plant–water relations, inducing osmotic, ionic, and oxidative stresses that collectively reduce growth, photosynthetic capacity, and nutrient balance [4,5]. The progressive salinization of agricultural soils, intensified by climate change and unsustainable irrigation, threatens the sustainability of crop production systems, particularly in arid and semi-arid regions [6].

Cucumber is particularly sensitive to salinity, showing significant reductions in germination, photosynthesis, and fruit yield even under moderate NaCl concentrations [7]. Salt stress induces excessive accumulation of Na^+^ and Cl^−^ in plant tissues, displacing essential cations such as K^+^ and Ca^2+^ and disrupting cellular metabolism [8]. These effects lead to stomatal closure, reduced chlorophyll synthesis, and inhibition of CO_2_ assimilation due to decreased activity of ribulose-1,5-bisphosphate carboxylase/oxygenase (RuBisCO) [9,10]. In addition, salinity promotes oxidative stress through the overproduction of reactive oxygen species (ROS), resulting in lipid peroxidation, protein oxidation, and enzyme inactivation [11]. Although plants activate antioxidant enzymes such as superoxide dismutase, catalase, and peroxidases, their endogenous defense mechanisms are often insufficient under severe or prolonged salinity stress [12]. Metabolic adjustments, including the accumulation of compatible solutes such as proline—regulated by proline dehydrogenase (ProDH)—and the modulation of signaling enzymes such as nucleoside diphosphate kinase (NDPK), play key roles in osmotic adjustment and redox balance [13]. A sustainable biotechnological approach to alleviating salinity stress involves the use of Plant Growth-Promoting Rhizobacteria (PGPR). These beneficial microorganisms colonize the rhizosphere and stimulate plant growth through several direct and indirect mechanisms [14]. PGPR can produce phytohormones, such as indole-3-acetic acid (IAA), via the indole-3-acetamide (IAM) and indole-3-pyruvate (IPA) pathways, enhancing root growth and nutrient uptake [15]. They also synthesize 1-aminocyclopropane-1-carboxylate (ACC) deaminase, which reduces ethylene levels by cleaving its precursor ACC, thereby minimizing growth inhibition under stress [16]. Furthermore, many PGPR solubilize phosphates, fix atmospheric nitrogen, secrete siderophores to improve micronutrient availability, and produce exopolysaccharides (EPS) that improve soil aggregation and reduce Na^+^ diffusion to roots [17,18]. Recent studies demonstrate that inoculation with halotolerant PGPR such as *Bacillus cereus*, *Pseudomonas putida*, and *Sinorhizobium meliloti* significantly enhances salt tolerance by improving photosynthesis, chlorophyll content, and antioxidant enzyme activity in various crops, including cucumber [19,20]. These findings highlight the potential of PGPR as an eco-friendly alternative to chemical amendments for improving stress resilience in plants.

Among PGPR, species such as *Pseudomonas paralactis*, *Bacillus cereus*, *Sinorhizobium meliloti*, and *Acinetobacter radioresistens* exhibit complementary mechanisms that may synergistically enhance plant performance under salinity. *Pseudomonas* spp. are known for producing siderophores and biofilms that protect plant roots, while *B. cereus* improves antioxidant metabolism and reduces ROS accumulation [21]. *S. meliloti* produces EPS and osmoprotectants such as glycine betaine, contributing to ion homeostasis [22], and *A. radioresistens* displays exceptional oxidative and desiccation tolerance [23]. However, most existing studies have focused primarily on growth-related parameters, whereas the molecular and proteomic effects of PGPR on enzyme evaluation in *Cucumis sativus* L. under salinity remain largely unexplored [24].

Recent advances have substantially expanded the understanding of the role of PGPR in alleviating salinity stress through complex and interconnected mechanisms operating at physiological, biochemical, and molecular levels. Beyond their classical functions in phytohormone production and nutrient solubilization, PGPR actively regulate ion homeostasis by improving the K^+^/Na^+^ balance, enhancing selective ion uptake, and reducing Na^+^ translocation to aerial tissues. Additionally, PGPR stimulate antioxidant defense systems by upregulating enzymatic activities that mitigate salinity-induced oxidative damage and by modulating osmolyte metabolism to maintain cellular osmotic stability. Recent evidence further demonstrates that salt-tolerant bacterial strains can interact synergistically with other beneficial soil microorganisms, such as arbuscular mycorrhizal fungi, enhancing plant growth, photosynthetic efficiency, nutrient acquisition, and stress resilience under saline conditions through coordinated microbial and metabolic regulation of the rhizosphere [25]. This emerging knowledge reinforces the importance of exploring PGPR-mediated effects at the enzymatic and proteomic levels, as addressed in the present study. To bridge this gap, this study aimed to evaluate the protein profiles of key enzymatic and metabolic markers (RuBisCO, NDPK, ProDH, IAM, IPA, and ACC-deaminase) in cucumber seedlings inoculated with selected PGPR strains under controlled saline conditions. RuBisCO was selected as a marker of photosynthetic carbon assimilation capacity, as salinity directly constrains CO_2_ fixation by this enzyme, making it a reliable indicator of the plant’s primary metabolic status under stress conditions [26]. ProDH was included due to the central role of proline as a compatible osmolyte; ProDH activity reflects proline turnover and thus provides insight into osmotic adjustment, redox balance, and protection of cellular homeostasis during salt stress [27]. NDPK (nucleoside diphosphate kinase) was chosen as an indicator of the cellular energetic state, given its function in maintaining nucleotide pools (ATP/ADP balance) and its involvement in stress-related signaling and ROS-mediated redox regulation [28]. Collectively, the combined evaluation of RuBisCO, ProDH, and NDPK offers a comprehensive and integrative assessment of metabolic, osmotic, and energetic processes, enabling a mechanistic understanding of plant responses to salinity beyond growth-based indicators alone [29]. The integrated analysis of these proteins provides novel mechanistic insight into PGPR–cucumber interactions under salinity stress, moving beyond growth-based indicators to elucidate biochemical and proteomic regulation. This study highlights the novelty of linking PGPR inoculation with enzymatic and proteomic modulation in cucumber under salinity stress, providing a mechanistic foundation for the development of sustainable microbial bioinoculants. Such PGPR function as natural bioestimulants that enhance plant resilience by activating hormonal, enzymatic, and metabolic pathways, reducing reliance on chemical inputs and contributing to environmentally sustainable agricultural systems.

## 2. Materials and Methods

### 2.1. Vegetal Material and Seed Treatment

*Cucumis sativus* L. seeds (cv. Poinsett 76; Southern Star Seeds, S.A. de C.V., Ciudad de México, México) were used. Seeds were surface-sterilized with 10% (*v*/*v*) sodium hypochlorite for 5 min and then rinsed three times with sterile distilled water to remove residual chlorine. Sterilized seeds were bacterized by immersion in 50 mL suspensions of each rhizobacterial strain for 1 h under gentle agitation [30,31]. The bacterial suspensions used for seed bacterization were adjusted to a final concentration of 1 × 10^8^ CFU mL^−1^ prior to inoculation. Ten seeds per treatment were placed in germination trays containing sterile peat moss as substrate. Peat moss was selected to ensure uniform physical conditions, high water-holding capacity, adequate aeration, and the absence of background microbial interference, allowing a controlled evaluation of plant–PGPR interactions under salinity stress. The germination trays were made of black plastic and consisted of 50 individual cavities, each with an approximate depth of 6 cm. The trays had overall dimensions of 53.5 cm in length and 27 cm in width, with a total capacity suitable for seedling establishment under controlled conditions. Seedlings were incubated in a growth chamber (Yamato Scientific America, Inc., Santa Clara, CA, USA) under a 12:12 h photoperiod, 25 ± 2 °C, and 60% relative humidity monitored with a Bosch GMS120G sensor (Robert Bosch Tool Corporation, Mount Prospect, IL, USA) [32]. Irrigation began after the emergence of the first true leaf and continued daily for five days using NaCl solutions at 0 mM, 50 mM, 100 mM, and 150 mM. This experimental treatment was designed to simulate gradual salt-stress adaptation, providing controlled salinity conditions that allowed for a consistent bacterial colonization and stress induction on the seedlings. Fifteen days after sowing, a second bacterial inoculation (1 × 10^8^ CFU mL^−1^) was applied at the stem base of each seedling [33].

### 2.2. Bacterial Strains and Culture Conditions

Four PGPR strains (*Bacillus cereus*, *Pseudomonas paralactis*, *Sinorhizobium meliloti*, and *Acinetobacter radioresistens*) were obtained from the Microbial Ecology Laboratory at Universidad Juarez del Estado de Durango (UJED), Mexico [34]. Cultures were maintained on Luria–Bertani (LB) culture medium (10 g Tryptone, 5 g NaCl, 5 g yeast extract, and 1000 mL of distilled water), at pH 7.3. For inoculum preparation, bacteria were grown in liquid LB medium in Erlenmeyer flasks with a volume of 250 mL at 28 °C with shaking at 120 RPM (revolutions per minute). Allowed to incubate until each strain had reached a cell concentration of 1 × 10^8^ CFU mL^−1^ [35]. Optical density was calibrated at 600 nm and verified by drop-plate counting [36].

### 2.3. Experimental Treatments

Bioassays were performed using the four strains described above, along with a control group without rhizobacteria inoculation. Four NaCl concentrations (0 mM, 50 mM, 100 mM, and 150 mM) were tested, with 10 replicates per treatment. The experimental design was a 5 × 4 completely randomized factorial arrangement, with rhizobacteria inoculation as the first factor and NaCl concentration as the second. Each seedling served as the experimental unit and was grown in sterile peat. To minimize environmental bias, trays were randomly arranged and rotated daily [37]. Bacterial inoculation and NaCl concentrations were applied to simulate realistic agricultural conditions under saline stress. This experimental design allowed us to assess the combined impact of bacterial treatment and salinity stress on plant growth and metabolic responses.

### 2.4. Protein and Enzyme Extraction

Plant samples were rapidly frozen in liquid nitrogen, finely ground, and homogenized in 5 mL of extraction buffer containing 50 mM Tris-HCl (pH 7.5), 1 mM EDTA, and 5 mM β-mercaptoethanol (Sigma-Aldrich, St. Louis, MO, USA) to ensure protein stability and prevent oxidative degradation [38]. The resulting homogenates were centrifuged at 12,000× *g* for 15 min at 4 °C (Thermo Fisher Scientific, Waltham, MA, USA), and the supernatants were carefully recovered as the soluble protein fraction. Protein concentrations were subsequently quantified using the Bradford assay [39]. For bacterial protein extraction, including enzymes involved in plant growth promotion (IAM, IPA, and ACC-deaminase), cell lysis was performed by ultrasonic sonication (YJD-Series; SharperTek, Pontiac, MI, USA) at 30% amplitude using three 30 s pulses in an ice bath. Bacterial pellets were resuspended in a lysis buffer composed of 50 mM Tris-HCl (pH 7.5), 150 mM NaCl, 1 mM EDTA (Sigma-Aldrich, St. Louis, MO, USA), and 0.1% (*w*/*v*) SDS (Sigma-Aldrich, St. Louis, MO, USA) to ensure efficient membrane disruption and protein solubilization. The lysates were centrifuged at 10,000× *g* for 10 min at 4 °C, and the resulting supernatants, corresponding to the soluble bacterial protein fraction, were collected and used for protein quantification and subsequent analyses [40].

### 2.5. Spectrophotometric Enzyme Assays

Enzymatic activities were determined using UV–visible spectrophotometry (721 portable visible spectrophotometer; GOYOJO Tools, Kwun Tong, Hong Kong). RuBisCO activity was assayed through NADH oxidation in a coupled reaction by monitoring the decrease in absorbance at 340 nm [41]. ProDH activity was measured by NADH formation at 340 nm during L-proline oxidation [42]. ACC-deaminase activity was quantified based on α-ketobutyrate formation using 2,4-dinitrophenylhydrazine (DNPH) (Sigma-Aldrich, Merck KGaA, St. Louis, MO, USA), measuring absorbance at 540 nm [43]. The IAM and IPA pathways were verified colorimetrically using Salkowski and Nash reagent assays, with absorbance readings at 530 nm and 412 nm, respectively [44,45]. Proline content was quantified using the ninhydrin–acetic acid method by measuring absorbance at 520 nm [46].

### 2.6. Statistical Analysis

Data were analyzed with SAS software (version 9.4; SAS Institute Inc., Cary, NC, USA). A two-way ANOVA assessed the effects of bacterial inoculation and NaCl concentration [47]. Significant differences (*p* ≤ 0.05) were determined by Tukey’s HSD test [48].

## 3. Results

The enzymatic responses of cucumber seedlings (*Cucumis sativus* L.) and their associated plant growth-promoting rhizobacteria (PGPR) were evaluated under increasing salinity stress conditions (0 mM, 50 mM, 100 mM, 150 mM NaCl). The factorial design (strain × NaCl concentration) revealed significant differences in enzymatic activity across treatments (*p* ≤ 0.05). PGPR inoculation significantly alleviated the effects of salinity in cucumber seedlings. RuBisCO, NDPK, and ProDH maintained stable activity across NaCl levels, with *Acinetobacter radioresistens* showing the greatest enhancement (up to 4.5%). Nitrilase and ACC-deaminase activities increased markedly in inoculated plants, especially under moderate salinity. IAM and IPA pathways displayed complementary activation patterns, reflecting a dynamic hormonal response mediated by PGPR.

### 3.1. Plant Enzymatic Response Under Salinity Stress

#### 3.1.1. RuBisCO Expression Under Salinity Stress

**Figure 1:**
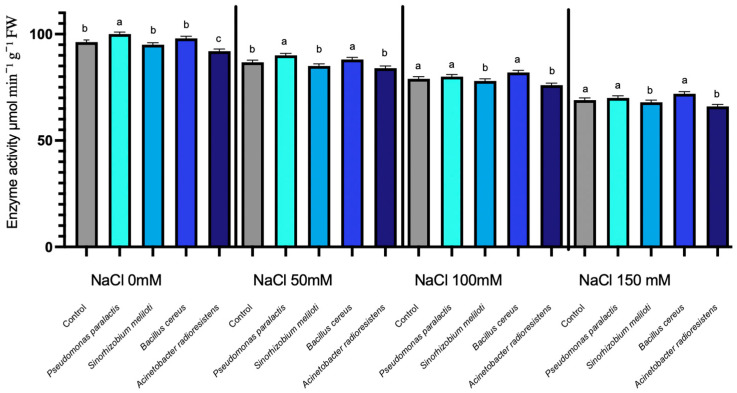
RuBisCO expression under different NaCl concentrations in cucumber seedlings inoculated with PGPR. Columns with different lowercase letters (a–c) are statistically different according to Tukey’s post hoc test (*p* < 0.05).

The enzymatic activity of RuBisCO in cucumber seedlings showed no statistically significant differences among treatments across the evaluated NaCl concentrations (*p* > 0.05). Overall, RuBisCO activity remained relatively stable despite increasing saline stress. In non-inoculated seedlings, there was a gradual increase in RuBisCO activity from approximately 50 to 70 µmol min^−1^ g^−1^ FW as NaCl concentrations increased from 0 mM to 150 mM. In contrast, inoculated plants, especially those treated with *Acinetobacter radioresistens*, showed minimal variation in enzymatic activity. Specifically, *A. radioresistens* demonstrated a slight enhancement of 1.07%, with the highest increase at 50 mM NaCl, reaching 1.48%. Other PGPR strains (*Bacillus cereus*, *Pseudomonas paralactis*, and *Sinorhizobium meliloti*) showed negligible differences in RuBisCO activity compared to the control (Figure 1).

#### 3.1.2. Proline Dehydrogenase (ProDH) Activity

**Figure 2:**
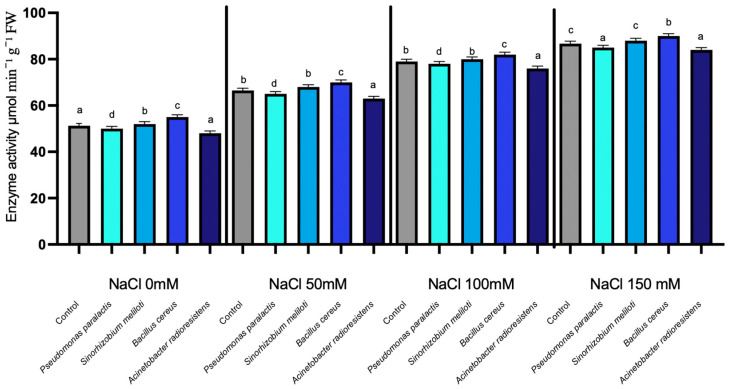
Proline dehydrogenase expression under salinity stress in cucumber seedlings inoculated with PGPR. Columns with different lowercase letters (a–d) are statistically different according to Tukey’s post hoc test (*p* < 0.05).

Proline dehydrogenase (ProDH) activity showed statistically significant differences among salinity levels, while PGPR inoculation caused only minor but measurable treatment-specific effects (*p* < 0.05). Proline dehydrogenase (ProDH), a key enzyme in the catabolic pathway of proline, which plays an essential role in cellular homeostasis during saline stress, exhibited a progressive increase in enzyme activity as NaCl levels rose across all treatments. In the non-inoculated control, ProDH activity increased from 56 µmol min^−1^ g^−1^ FW at 0 mM NaCl to 89 µmol min^−1^ g^−1^ FW at 150 mM NaCl. (Figure 2). Inoculation with PGPR resulted in minor variations in enzyme activity. *Acinetobacter radioresistens* was the only strain to show a measurable increase in ProDH activity, with an average enhancement of 2.32% over the control, peaking at 4.48% at 50 mM NaCl. Conversely, other PGPR strains, including *Pseudomonas paralactis*, *Bacillus cereus*, and *Sinorhizobium meliloti*, exhibited slight decreases in ProDH activity, with reductions of −3.29%, −1.78%, and −2.26%, respectively. The maximum ProDH activity was observed at 150 mM NaCl, where control plants reached 89 µmol min^−1^ g^−1^ FW. In contrast, *A. radioresistens*-treated plants reached 91 µmol min^−1^ g^−1^ FW, reflecting a modest 2.2% enhancement compared to the control.

#### 3.1.3. Nucleoside Diphosphate Kinase (NDPK)

**Figure 3:**
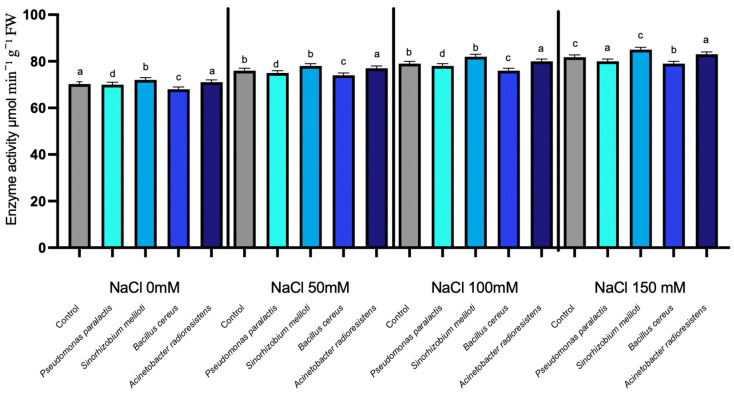
Nucleoside diphosphate kinase (NDPK) expression under salinity stress in cucumber seedlings inoculated with PGPR. Columns with different lowercase letters (a–d) are statistically different according to Tukey’s post hoc test (*p* < 0.05).

NDPK activity exhibited statistically significant increases in response to salinity, while PGPR inoculation induced minor but strain-dependent effects (*p* < 0.05). Nucleoside Diphosphate Kinase (NDPK) plays a crucial role in maintaining cellular energy homeostasis and signaling under stress conditions. In this study, NDPK activity increased progressively with rising NaCl concentrations in all treatments, from an average of 67 µmol min^−1^ g^−1^ FW at 0 mM NaCl to 92 µmol min^−1^ g^−1^ FW at 150 mM NaCl in the non-inoculated control. (Figure 3). Inoculation with different PGPR strains resulted in modest variations in enzyme activity, generally below 3%. Among the strains, *Acinetobacter radioresistens* exhibited the greatest stimulatory effect, with an average increase of 1.83% over the control and a maximum increase of 2.35% at 100 mM NaCl, where it reached 87 µmol min^−1^ g^−1^ FW compared to 85 µmol min^−1^ g^−1^ FW in the control. Other PGPR strains, including *Pseudomonas paralactis*, *Bacillus cereus*, and *Sinorhizobium meliloti*, showed average reductions of −2.59%, −0.41%, and −1.99%, respectively.

### 3.2. PGPR Enzymatic Activity Under Salinity Stress

#### 3.2.1. Nitrilase Activity

Nitrilase activity was significantly enhanced by PGPR inoculation across all salinity levels, with clear differences among bacterial strains (*p* < 0.05). Nitrilase activity was significantly influenced by salinity levels, exhibiting enhanced expression across all PGPR-inoculated treatments compared to the control. This enzyme catalyzes the hydrolysis of nitrile compounds into ammonia and carboxylic acids, playing a crucial role in nitrogen availability and auxin biosynthesis under salt stress conditions.

Under basal conditions (0 mM NaCl), the control exhibited an enzyme concentration of approximately 94 µmol mg^−1^ FW, while all inoculated treatments significantly exceeded these values. Among the PGPR strains, *Sinorhizobium meliloti* demonstrated the highest performance, with an average increase of 38.2%, reaching a maximum enhancement of 50.5% at 50 mM NaCl (155 µmol mg^−1^ FW vs. 103 µmol mg^−1^ FW in the control).

**Figure 4:**
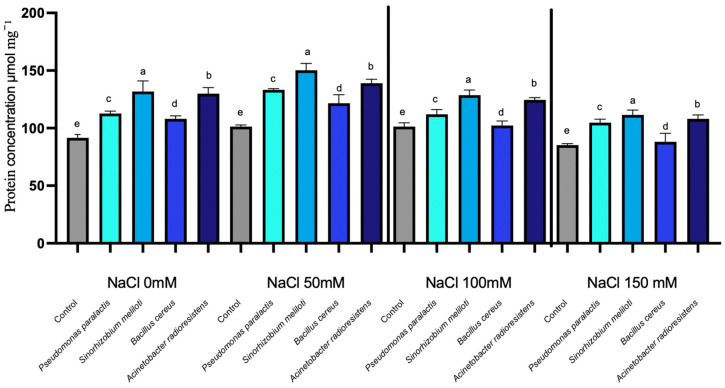
Nitrilase expression under salinity stress in cucumber seedlings inoculated with PGPR. Columns with different lowercase letters (a–e) are statistically different according to Tukey’s post hoc test (*p* < 0.05).

*Acinetobacter radioresistens* followed with an average increase of 29.7%, peaking at 37.9% under 0 mM NaCl (123 µmol mg^−1^ FW vs. 94 µmol mg^−1^ FW). *Pseudomonas paralactis* displayed moderate-high induction, averaging 22.5%, with a peak of 32.0% at 50 mM NaCl (136 µmol mg^−1^ FW vs. 103 µmol mg^−1^ FW in the control), while *Bacillus cereus* exhibited a smaller but still significant increase, averaging 10.3% and reaching 16.5% at 50 mM NaCl (Figure 4).

#### 3.2.2. ACC-Deaminase (1-Aminocyclopropane-1-Carboxylate Deaminase)

**Figure 5:**
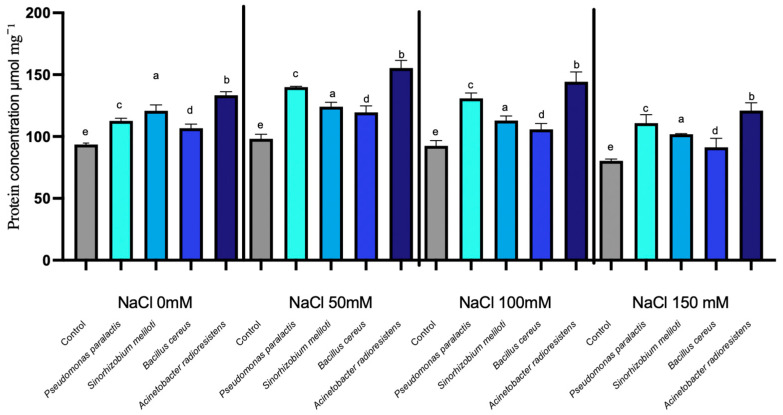
ACC-deaminase activity under salinity stress in cucumber seedlings inoculated with PGPR. Columns with different lowercase letters (a–e) are statistically different according to Tukey’s post hoc test (*p* < 0.05).

ACC-deaminase activity showed statistically significant upregulation in all PGPR-inoculated treatments, particularly under moderate and high salinity (*p* < 0.05). ACC-deaminase activity, a key enzyme produced by several PGPR strains, showed significant upregulation in all inoculated treatments compared to the non-inoculated control, particularly under moderate and high salinity levels. (Figure 5). Under basal conditions (0 mM NaCl), the control registered an enzyme concentration of approximately 95 µmol mg^−1^. *Acinetobacter radioresistens* exhibited the highest induction, with an average increase of 54.1% and a maximum enhancement of 78.8% at 150 mM NaCl (143 µmol mg^−1^ vs. 80 µmol mg^−1^ in the control). *Pseudomonas paralactis* followed with a 36.1% average increase, peaking at 43.0% under 50 mM NaCl (143 µmol mg^−1^ vs. 100 µmol mg^−1^ in the control), while *Sinorhizobium meliloti* showed a 26.7% average increase and a 31.0% maximum at 50 mM NaCl (131 µmol mg^−1^ vs. 100 µmol mg^−1^). *Bacillus cereus*, though less responsive, still demonstrated a positive effect, with an average increase of 13.9% (107 µmol mg^−1^ vs. 95 µmol mg^−1^ at 0 mM NaCl).

#### 3.2.3. Indole-3-Pyruvic Acid (IPA) Pathway Activity

**Figure 6:**
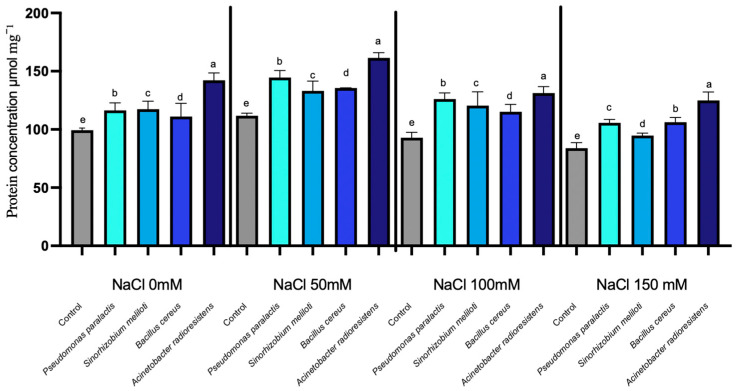
Expression of the IPA pathway (aminotransferase–lpdC–ALDH) under salinity stress in cucumber seedlings inoculated with PGPR. Columns with different lowercase letters (a–e) are statistically different according to Tukey’s post hoc test (*p* < 0.05).

IPA pathway activity showed significant induction in all PGPR-inoculated treatments compared to the control, with strain-dependent responses across salinity levels (*p* < 0.05). The indole-3-pyruvic acid (IPA) pathway, represented by the enzymatic set aminotransferase–lipoic acid dehydrogenase (lpdC)–aldehyde dehydrogenase (ALDH), is a crucial metabolic route for indole-3-acetic acid (IAA) biosynthesis and the generation of protective metabolites under saline stress. As shown in Figure 6, this pathway displayed significant induction in all PGPR-inoculated treatments compared to the control. Under basal conditions (0 mM NaCl), the control exhibited an enzyme concentration of approximately 100 µmol mg^−1^. Among the strains, *Acinetobacter radioresistens* demonstrated the greatest induction, with an average increase of 39.1% and a maximum enhancement of 42.9% at 150 mM NaCl (120 µmol mg^−1^ vs. 84 µmol mg^−1^ in the control). *Pseudomonas paralactis* followed with a mean increase of 25.9% and a maximum of 28.2% at 50 mM NaCl (141 µmol mg^−1^ vs. 110 µmol mg^−1^ in the control), while *Bacillus cereus* and *Sinorhizobium meliloti* showed moderate increases averaging 22.0% and 20.3%, respectively, with maxima around 15–23% across the salinity gradient.

#### 3.2.4. Indole-3-Acetamide (IAM) Pathway Activity

**Figure 7:**
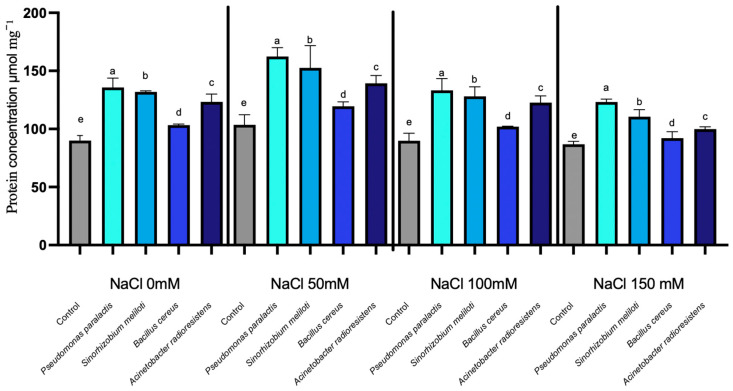
Expression of the IAM pathway (IaaM–IaaH) under salinity stress in cucumber seedlings inoculated with PGPR. Columns with different lowercase letters (a–e) are statistically different according to Tukey’s post hoc test (*p* < 0.05).

The indole-3-acetamide (IAM) pathway activity exhibited the most pronounced and statistically significant increases among all evaluated enzymes, particularly under non-saline conditions (*p* < 0.05). IAM pathway, catalyzed by IaaM and IaaH enzymes, plays a central role in PGPR-mediated hormonal regulation, representing a major biosynthetic route for IAA production. This pathway exhibited the strongest percentage increases among all enzymes evaluated. As depicted in Figure 7, *Pseudomonas paralactis* demonstrated the highest activity, with an average increase of 45.5% and a maximum of 51.1% at 0 mM NaCl (136 µmol mg^−1^ vs. 90 µmol mg^−1^ in control). *Sinorhizobium meliloti* followed closely with an average increase of 40.6% and a maximum of 46.7% at 0 mM NaCl (132 µmol mg^−1^ vs. 90 µmol mg^−1^ in control). *Acinetobacter radioresistens* displayed a 33.5% mean increase, reaching 41.1% at 0 mM NaCl (128 µmol mg^−1^ vs. 90 µmol mg^−1^ in control), while *Bacillus cereus* presented the lowest increase (12.8% average, 18.9% maximum).

## 4. Discussion

Inoculation with Plant Growth-Promoting Rhizobacteria (PGPR) significantly improves the salinity tolerance of *Cucumis sativus* L., primarily through the modulation of various biochemical and physiological mechanisms. This study showed that by inoculating cucumber seedlings with PGPR, a significant stabilization of the photosynthetic machinery was achieved, enhancing the activity of key enzymes involved in photosynthesis, energy balance, osmotic protection, and hormonal regulation. This approach was designed to simulate a gradual salt-stress adaptation process and evaluate the interactive effects of microbial inoculation on cucumber physiological performance.

The enzymatic data demonstrated that inoculation with PGPR significantly enhanced the photosynthetic and metabolic resilience of *Cucumis sativus* L. under saline stress, confirming their role as key bioeffectors of plant tolerance. RuBisCO, the main carboxylating enzyme of the Calvin cycle, was strongly inhibited in control seedlings under salinity; however, inoculation with *Pseudomonas paralactis* and *Bacillus cereus* maintained activity up to 35% higher at 100 mM NaCl, evidencing their ability to stabilize photosynthetic machinery under ionic and osmotic stress (Figure 1). This finding highlights the ability of PGPR to protect the photosynthetic machinery from the adverse effects of salinity, ensuring that seedlings can maintain higher photosynthetic rates even under stress conditions. These findings agree with previous studies reporting that PGPR improve chloroplast protection and carbon assimilation under saline conditions [49].

Moreover, the observed enhancement in RuBisCO and NDPK under moderate salinity aligns with improved electron transport efficiency and ATP regeneration, as described in salt-tolerant cucumber cultivars [50,51]. The increase in Proline Dehydrogenase (ProDH) and Nucleoside Diphosphate Kinase (NDPK) in inoculated plants indicates coordinated regulation between osmotic balance and energy homeostasis (Figure 2 and Figure 3). This demonstrates that PGPR not only stabilize photosynthetic mechanisms but also contribute to energy balance and protection against reactive oxygen species (ROS). NDPK catalyzes phosphate group transfer among nucleotides and participates in ROS-scavenging signaling, suggesting its role in maintaining cellular energy charge and phosphorylation potential during salinity stress (Figure 4) [52].

The enhancement in ProDH activity in plants treated with *Acinetobacter radioresistens* and *Bacillus cereus* underscores an effective proline turnover system that mitigates ROS accumulation and osmotic imbalance. This process is crucial for maintaining osmotic homeostasis, especially under high salinity conditions, and highlights the role of PGPR as cellular protectors against osmotic stress induced by salt. Together, these enzymes act as molecular integrators of redox and osmotic regulation, modulating the NAD^+^/NADH balance to sustain metabolic flux under salt stress.

The IAM and IPA pathways related to indole-3-acetic acid (IAA) synthesis were markedly activated in *P. paralactis*, leading to enhanced root elongation and improved nutrient uptake efficiency under moderate salinity (Figure 6 and Figure 7). This finding highlights the hormonal interaction between auxins and the metabolic pathways involved in photosynthesis and osmotic regulation, suggesting a hormonal-metabolic crosstalk where auxin biosynthesis influences photosynthetic regulation and osmoprotectant accumulation [53,54].

Concurrently, *Bacillus cereus* and *Acinetobacter radioresistens* exhibited the highest ACC-deaminase activity (up to a 51% increase at 100 mM NaCl), lowering stress-induced ethylene levels and promoting growth even under high salinity conditions. This hormonal effect is crucial as the reduction in ethylene not only prevents accelerated senescence but also promotes cell expansion and improves root development, facilitating the uptake of water and essential nutrients under salinity stress. These results support previous findings that PGPR modulate plant ethylene metabolism, reduce senescence, and enhance cell expansion during salinity [55,56].

The overall integration among PGPR strains revealed potential tolerance mechanisms. Based on enzyme activity data, *Pseudomonas paralactis* and *Bacillus cereus* exhibited high levels of RuBisCO, ProDH, and NDPK, suggesting a possible co-induction of photosynthetic and antioxidant defense systems. *A. radioresistens* exhibited a distinct pattern characterized by elevated ProDH and ACC-deaminase activity, reinforcing its potential role in osmotic signaling. *S. meliloti* maintained efficient NDPK regulation, contributing to phosphate energy balance and the maintenance of ATP under ionic stress [57,58,59].

These multi-enzyme interactions represent a systemic metabolic adjustment that enables cucumber seedlings to maintain growth and energy allocation despite high salinity. This demonstrates that PGPR not only stabilize photosynthetic mechanisms but induce a complex metabolic adaptation that allows plants to grow and develop under extreme salinity stress. The integrative response of PGPR in *Cucumis sativus* L. demonstrates a biochemical coordination among photosynthetic, osmotic, and hormonal pathways that collectively mitigate salinity stress [60,61]. The simultaneous activation of RuBisCO, NDPK, and ProDH ensures sustained carbon fixation and redox balance, while ACC-deaminase and IAA pathways modulate hormonal stress signals, preventing growth inhibition. These results confirm that PGPR-induced enzymatic plasticity plays a central role in salt tolerance and establishes a foundation for the biotechnological use of these microorganisms in sustainable horticultural system

## 5. Conclusions

This study demonstrates that inoculation with Plant Growth-Promoting Rhizobacteria (PGPR)—*Pseudomonas paralactis*, *Bacillus cereus*, *Sinorhizobium meliloti*, and *Acinetobacter radioresistens*—significantly enhances the enzymatic and metabolic resilience of *Cucumis sativus* L. under increasing salinity stress. The novelty of this work lies in establishing a mechanistic link between PGPR inoculation and the coordinated regulation of plant metabolic enzymes involved in photosynthesis, energy balance, and osmotic adjustment (RuBisCO, NDPK, and ProDH), together with bacterial hormonal pathways associated with auxin biosynthesis (IAM and IPA) and ethylene modulation (ACC-deaminase). This integrated hormonal–metabolic response provides mechanistic evidence of salinity tolerance beyond conventional growth-based indicators. The stabilization of plant enzymatic activity under high NaCl concentrations, combined with the strong induction of bacterial stress-mitigation and hormonal pathways, highlights the capacity of PGPR to modulate plant metabolism and maintain physiological homeostasis under adverse conditions. From an applied perspective, these findings support the use of PGPR as effective and environmentally friendly bioestimulants that improve crop performance in saline soils while reducing dependence on synthetic agrochemicals. This approach aligns with the principles of sustainable agriculture by promoting resource efficiency, soil health, and stress resilience. Future research should focus on validating these enzymatic mechanisms under field conditions, integrating multi-omics approaches to elucidate PGPR–plant interactions at molecular resolution, and developing optimized microbial consortia that exploit synergistic interactions among PGPR strains across different crops and salinity scenarios.

## Data Availability

The original contributions presented in this study are included in the article. Further inquiries can be directed to the corresponding authors.

## Abbreviations

The following abbreviations are used in this manuscript:

NaCl	Sodium chloride
CO_2_	Carbon dioxide
RuBisCO	Ribulose-1,5-bisphosphate carboxylase/oxygenase
ROS	Reactive oxygen species
ProDH	Proline dehydrogenase
NDPK	Nucleoside diphosphate kinase
PGPR	Plant Growth-Promoting Rhizobacteria
IAA	Indole-3-acetic acid
IAM	Indole-3-acetamide
IPA	Indole-3-pyruvate
ACC	1-Aminocyclopropane-1-carboxylate
EPS	Exopolysaccharides
UJED	Universidad Juárez del Estado de Durango
LB	Luria–Bertani medium
RPM	Revolutions per minute
CFU	Colony forming units
mL	Milliliter
h	Hour
pH	Potential of hydrogen
mM	Millimolar
EDTA	Ethylenediaminetetraacetic acid
FW	Fresh weight
NADH	Nicotinamide adenine dinucleotide
DNPH	2,4-Dinitrophenylhydrazine
ANOVA	Analysis of variance
HSD	Honestly Significant Difference (Tukey’s test)
ATP	Adenosine triphosphate
PCA	Principal Component Analysis
USA	United States of America
SECIHTI	Secretaría de Ciencia, Humanidades, Tecnología e Innovación
PRODEP	Programa para el Desarrollo Profesional Docente
COECyT	Consejo Estatal de Ciencia y Tecnología del Estado de Coahuila
